# Exosomes from adipose-derived mesenchymal stem cells prevent cardiomyocyte apoptosis induced by oxidative stress

**DOI:** 10.1038/s41420-019-0159-5

**Published:** 2019-03-19

**Authors:** Zhi Liu, Yueqiao Xu, Yungao Wan, Jing Gao, Yanyan Chu, Jing Li

**Affiliations:** 1 Department of Cardiology, Xuanwu Hospital, Capital Medical University, Beijing, China; 2 Department of Neurosurgery, Xuanwu Hospital, Capital Medical University, Beijing, China

## Abstract

Exosomes from bone marrow stem cells or cardiac progenitor cells can reduce apoptosis in myocardial cells after ischemia and reperfusion injury. However, there is little known about the effects of exosomes from adipose-derived stem cells (ADSCs), which are more abundant and have a lower risk of side effects. The aim of this study was to characterize exosomes from ADSCs and evaluate their cardioprotective actions against ischemia reperfusion injury. The exosomes were isolated from ADSCs and analyzed by protein marker expression, transmission electron microscopy, and nanoparticle tracking analysis. The ADSC-exosomes were then used for ex vivo investigation of the cardioprotective effects on cardiomyocytes after exposure to oxidative stress. Exosomes from ADSCs exhibited a diameter of 150 nm and expressed the marker proteins, CD9 and CD29. ADSC-exosomes had no effect on proliferation of untreated cardiomyocytes. In contrast, ADSC-derived exosomes reduced apoptosis in myocardial cells subjected to oxidative stress. This study confirms that exosomes originating from ADSCs can protect cardiomyocytes from oxidative stress.

## Introduction

Acute reperfusion is the best treatment for acute myocardial infarction (AMI). However, ischemic reperfusion injury induces oxidative stress and inflammation, leading to myocardial cell apoptosis, myocardial remodeling, and decreased cardiac function^1,2^. Currently, there is no effective clinical therapy for ischemia reperfusion injury^3^. Cell therapies were developed in the hope that stem cells could proliferate and differentiate into new myocardial cells to improve myocardial function after myocardial infarction^3,4^. However, <1% of stem cells reach the site of injury and differentiate in cariomyocytes^5^.

Exosomes may mediate much of the therapeutic benefit of stem cells^6–9^. Exosomes are lipid bilayer membrane vesicles formed by stem cells through the process of endocytosis, fusion, and efflux. Exosomes can alter the behavior of recipient cells and improve the cardiac microenvironment after AMI. Exosomes can ferry microribonucleic acid (miRNA) and proteins, facilitating intercellular communication through target cell internalization, receptor–ligand interactions, or lipid–membrane fusion^10,11^. Exosomes of various mesenchymal stem cells (MSCs) have been shown to restore bioenergetics, alleviate inflammatory and oxidative stress, and improve vascular generation^12–16^. As shown in animal and human models of myocardial ischemia reperfusion injury, exosomes can inhibit myocardial apoptosis, narrow the scope of myocardial infarction, preserve left ventricular geometry, and improve cardiac function^17–21^. However, exosomes secreted by adipose-derived stem cells (ADSCs) have not been fully characterized.

We hypothesized that endogenous exosomes communicate signals to the heart to protect it against ischemia and reperfusion injury. Oxidative stress is crucial to the development of ischemia-reperfusion injury. Oxidative stress activates reactive oxygen species (ROS) signaling, leading to cell death. In many ischemia-reperfusion injury studies, cardiomyocytes were exposed to H_2_O_2_^22,23^. H_2_O_2_-induced oxidative stress can reduce viability and increase apoptosis of cardiomyocytes. The aim of our study was to examine the effects of exosomes on cardiomyocytes under oxidative stress. We purified, characterized, and quantified exosomes from ADSCs of mice. Our results show that exosomes from ADSCs protect against oxidative stress injury in vitro.

## Materials and methods

### Cell culture

We purchased mouse ADSCs from Cyagen Biosciences Inc. (cat. MUBMD-01001). ADSCs were routinely cultured in DMEM/F12 (1:1) medium containing 100 U/mL penicillin, 100 μg/mL streptomycin, and 10% fetal bovine serum (FBS). The M6200 mouse cardiomyocyte cell line (MCM) was obtained from the Chinese Science Academy Cell Bank (Shanghai, China). MCM cells were cultured in high-glucose DMEM medium with 100 μg/mL streptomycin, 100 μg/mL penicillin (Gibco, NY, USA), and 10% FBS in a humidified incubator at 37 °C with 5% CO_2_.

### Identification of ADSCs

The ADSCs from passage 3 were digested with 0.25% trypsin, and 1 × 10^6^ cells were incubated with the following antibodies: Anti-Human CD34 FITC (1:1000; eBioscience), Anti-Human CD29 APC (1:1000; eBioscience), and FITC Mouse Anti-Human CD44 (1:1000; BD Pharmingen). Cell purity was determined by surface marker detection using flow cytometry^24^.

### CCK-8 assay for growth curve of ADSCs and proliferation

The ADSC suspension was collected and added to 96-well plates, 100 µL per well. The culture plates were placed in a 37 °C incubator and three wells were chosen randomly at 0, 2, 4, 6, and 8 days. The cells in the selected wells were incubated with 10 µL of Counting Kit-8 (CCK-8) for an additional 1 h. The optical density (OD) of each well at a wavelength of 450 nm was measured with a microplate reader (Bio-Rad, USA) to generate a cell growth curve. Each test was performed in triplicate.

### Flow-cytometric analysis of apoptosis

The effect of ADSCs and exosomes on cell apoptosis was determined using an Annexin V-FITC Apoptosis Detection Kit (Beyotime) and flow cytometry. Co-culture experiments were performed using transwell chamber inserts with 0.4 μm pores. MCM cells were seeded in the bottom chambers and ADSCs were seeded in the top chambers. After 200 µM H_2_O_2_ treatment for 4 h, 5 × 10^4^ MCM cells were suspended in 195 μl Annexin V-FITC-binding buffer. MCM cells were co-cultured with ADSCs or not, and then incubated for 10 min at room temperature in the dark after the addition of 5 μl Annexin V-FITC and 5 μl PI staining solution. Annexin V-FITC binding and PI staining were detected using flow cytometry and analyzed with FACSDiva (Becton-Dickinson) software. The experiments were performed independently three times^25^.

### Exosome purification

Exosomes secreted by ADSCs were purified from conditioned medium as described previously^26^. Briefly, 10 mL of culture medium (ADSCs) with 10% exosome-depleted FBS was added to ADSC cell cultures in 10 cm dishes. After 24 h, medium was centrifuged at 1000 RPM for 10 min and the supernatant was passed through 0.22 μm filters to remove dead cells and debris. The high purity exosomes were extracted according to the MagCapture^TM^ Exosome Isolation Kit PS (Wako, cat. 293-77601). Tim4 protein solidified magnetic beads were used to bind phosphatidyl serine (PS) on the surface of extracellular vesicles in the presence of metal ions, then neutral elution buffer containing EDTA was used to elute the exosome. By using the PS affinity method, isolation of exosomes with high purity and integrity was achieved. The exosome fraction protein content was assessed by the bicinchoninic acid (BCA) assay. We measured the exosome particle size (diameter) and concentration using nanoparticle tracking analysis (NTA).

### Electron microscopy

For transmission electron microscopy (TEM), 10 μl of PBS containing exosomes was placed on formvar carbon-coated 200-mesh copper electron microscopy grids and incubated for 1 min at room temperature^21^. For negative staining, 3% (w/v) aqueous phosphotungstic acid (pH 6.8) was applied onto the grid immediately after water removal and then removed with filter paper after 30 s. The grid was washed with double-distilled water and allowed to semi-dry at room temperature before being imaged by TEM.

### Western blotting

Exosomal protein content was measured using a BCA assay. Total protein (40 μg) was electrophoresed using 10% SDS–PAGE, and transferred to PVDF membranes (Millipore, USA). Membranes were blocked for 1 h at RT with 5% non-fat milk. Then membranes were incubated overnight at 4 °C with the primary antibody (rabbit anti-CD29, 1:1000, Proteintech or mouse anti-CD63, 1:1000, Abcam) diluted in TBST with 5% BSA. Membranes were washed with TBST, followed by incubation for 1 h at room temperature with either horseradish-peroxidase (HRP)-conjugated goat anti-rabbit secondary antibodies or HRP-conjugated goat anti-mouse (1:1000, Beyotime). The bands were visualized using ECL detection.

### Exosome labeling with PKH26

Purified exosomes derived from ADSCs were labeled using PKH26 red fluorescent labeling kits (Sigma-Aldrich) according to the manufacturer’s instructions. The excess dye was removed by adding 1% BSA solution. The samples were transferred to cell culture media. Exosomes labeled with PKH26 were incubated with the MCM cells for 0, 4, 8, and 24 h. The exosomes were removed from the MCM cells by washing the cells three times with PBS. The MCM cells were then incubated with 4% paraformaldehyde solution for 15 min, followed by three more washes. The washed membrane was mounted on a glass slide with mounting medium containing DAPI. The slides were analyzed using confocal microscopy.

### Statistical analysis

Data are expressed as mean ± SD and analyzed with SPSS 17.0 software. A *t*-test was used to evaluate the differences among groups. A value of *P* < 0.05 was considered to be statistically significant.

## Results

### Phenotypic characterization and anti-apoptotic effects of ADSCs

**Fig. 1:**
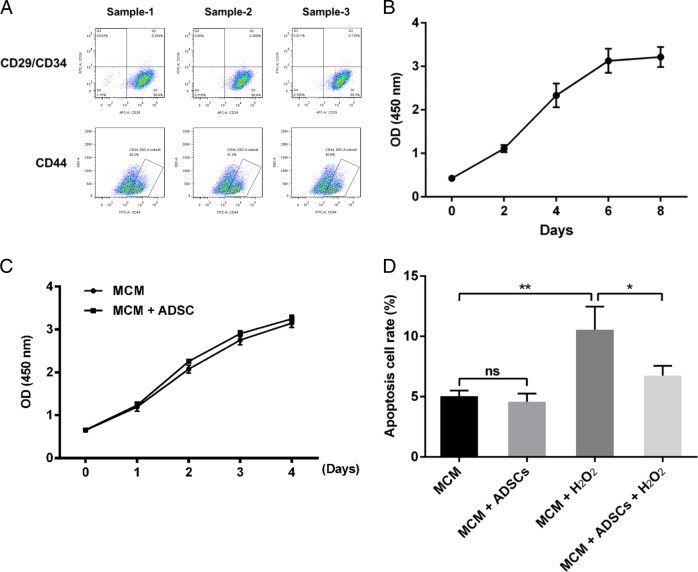
Phenotypic characterization and anti-apoptotic effects of ADSCs. **a** Samples of the flow cytometric dot plot for analysis of ADSCs markers. **b** Growth curves for adipose-derived stem cells. The cell density of P0, P2, P4, P6, and P8 were reflected by the OD (450 nm) value using a CCK8 assay. **c** CCK8 assay for the proliferation of MCM cultured alone or co-cultured with ADSCs. **d** The percentage of apoptosis cell rates from flow cytometry analysis results. Compared with control (MCM), compared with MCM treated with H_2_O_2_ group and MCM+ADSCs. ** *P* < 0.01; * *P* < 0.05; ns, no significance

ADSCs were cultured to passage 3. Phenotypic analysis via flow cytometry demonstrated that the ADSCs were positive for the MSC markers, CD29 and CD44, and negative for CD34 (Fig. 1a). Growth curves were generated to examine the proliferation of ADSCs. The growth curves for passages, P0, P2, P4, P6, and P8, were typically sigmoidal (Fig. 1b).

To assess the effects of ADSCs on cardiomyocytes, MCM cells were co-cultured with ADSCs for 24 h and then subjected to acute H_2_O_2_ oxidative stress (4 h). Co-culture with ADSCs resulted in almost no variation in the proliferation of untreated MCM cells (Fig. 1c). As shown in Fig. 1d, the rate of apoptosis for MCM cells was significantly increased after H_2_O_2_ treatment compared to untreated control cells, and ADSCs effectively suppressed this apoptosis in MCM cells.

### Characterization of ADSC-exosomes

**Fig. 2:**
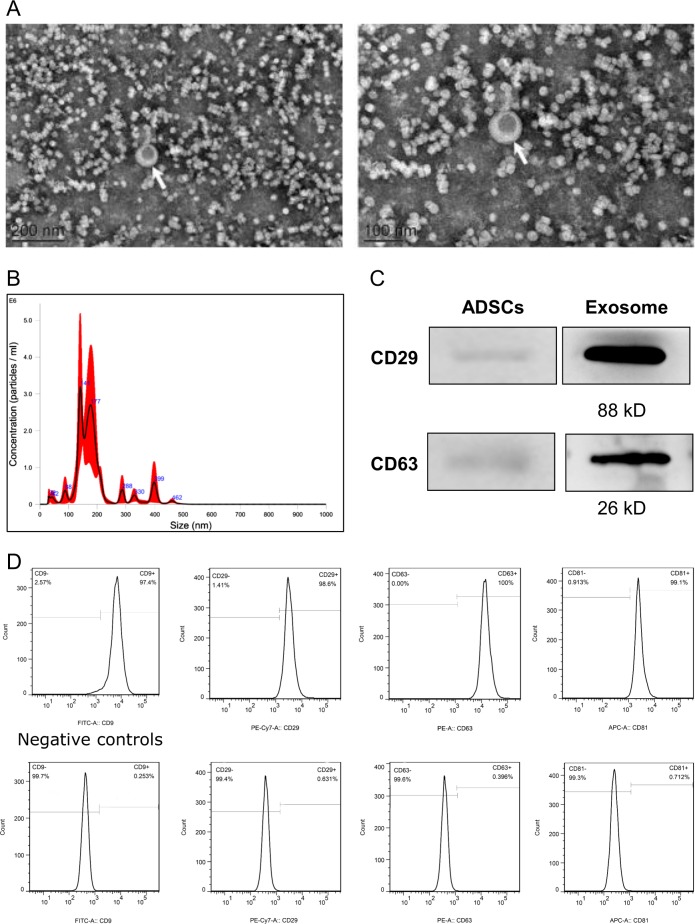
Characterization of ADSC-exosomes. **a** Electron micrograph image of ADSC-derived exosomes (arrow indicates exosomes). The image shows a mass of round-shaped vesicles. Scale bar = 200 nm. **b** Particle size distribution in purified pellets consistent with size range of exosomes (average size 150 nm) measured by nanoparticle tracking analysis (NTA). **c** Western blot results demonstrate the expression of CD29 and CD63 in exosomes derived from ADSCs. **d** FACS analysis of CD29 and CD63 expressed on the surface of ADSCs

Exosomes were readily detectable in media from ADSCs. Morphological analysis of the ADSC-exosomes using electron micrography demonstrated grape-like nanoparticle exosomes (Fig. 2a). The size of exosomes structures was determined using the nanoparticle tracking analyzer for hydrodynamic particle size (Fig. 2b). The pellets consisted of particles with an average size of 150 nm in diameter, consistent with the characteristic size range of exosomes (Fig. 2b). Western blot analysis of ADSC-exosomes revealed the presence of exosome markers CD63 and CD29 (Fig. 2c). FACS analysis demonstrate that markers CD29 and CD63 are expressed on the surface of the ADSCs (Fig. 2d).

### Exosome labeling and uptake by MCM cells

**Fig. 3:**
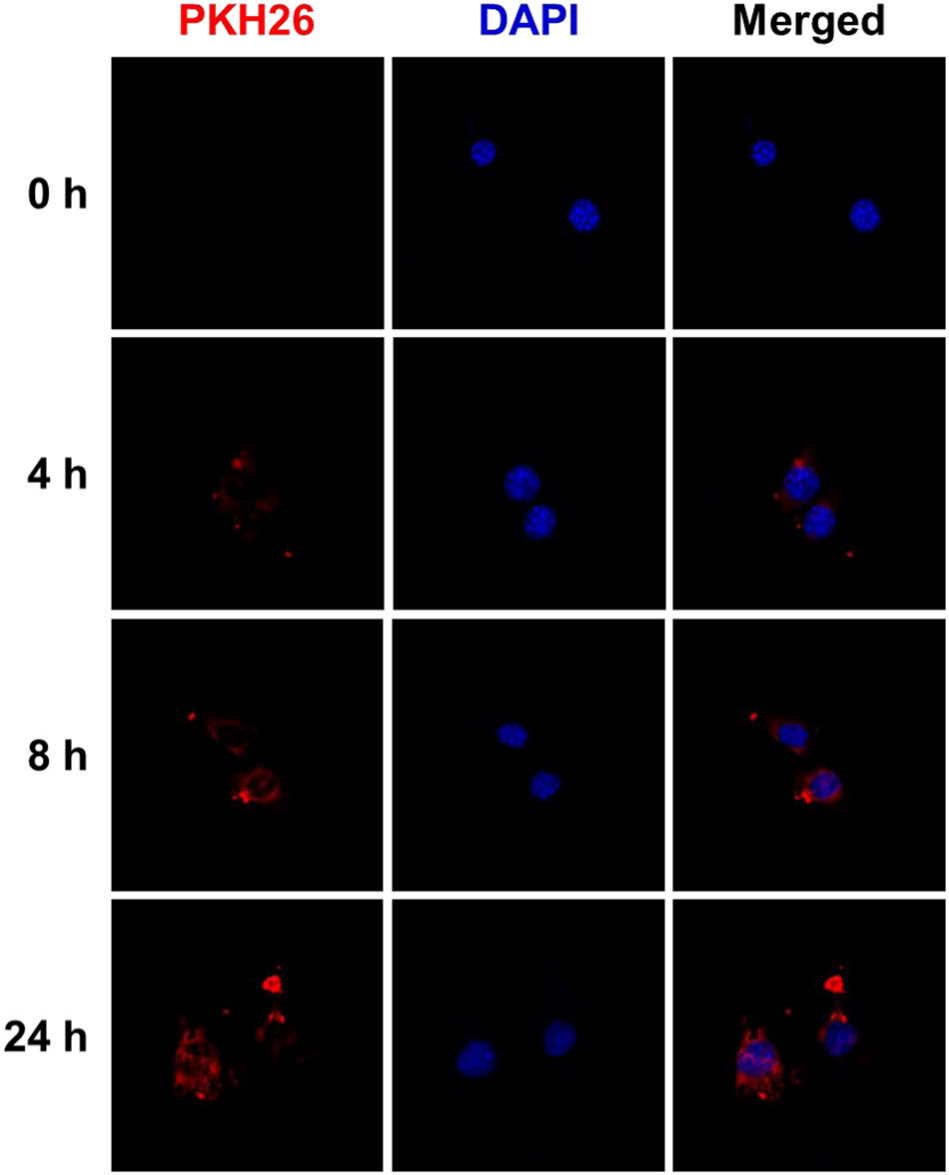
Time course of cellular uptake of exosomes

To determine whether ADSC-exosomes can be efficiently taken up by cardiomyocytes in vitro, we labeled ADSC-exosomes with PKH26, a fluorescent cell linker compound that is incorporated into the cell membrane by selective partitioning. The nuclei (blue) were counterstained with DAPI. The labeled exosomes were then incubated with MCM cells, which were subsequently examined for fluorescence at varying time points. Our results show that the PKH26-labeled exosomes were taken up by MCM cells within 4 h after the exosomes were added to the culture medium. Red fluorescence signals were detected in the MCM cells over time (Fig. 3). By 24 h, nearly every MCM cell exhibited red fluorescence. These finding demonstrate that ADSC-exosomes are readily taken up by MCM cells. Thus, exosomes can be used therapeutically to modulate cardiomyocyte function.

### Anti-apoptotic effects of ADSC-exosomes on MCM cells under oxidative stress

**Fig. 4:**
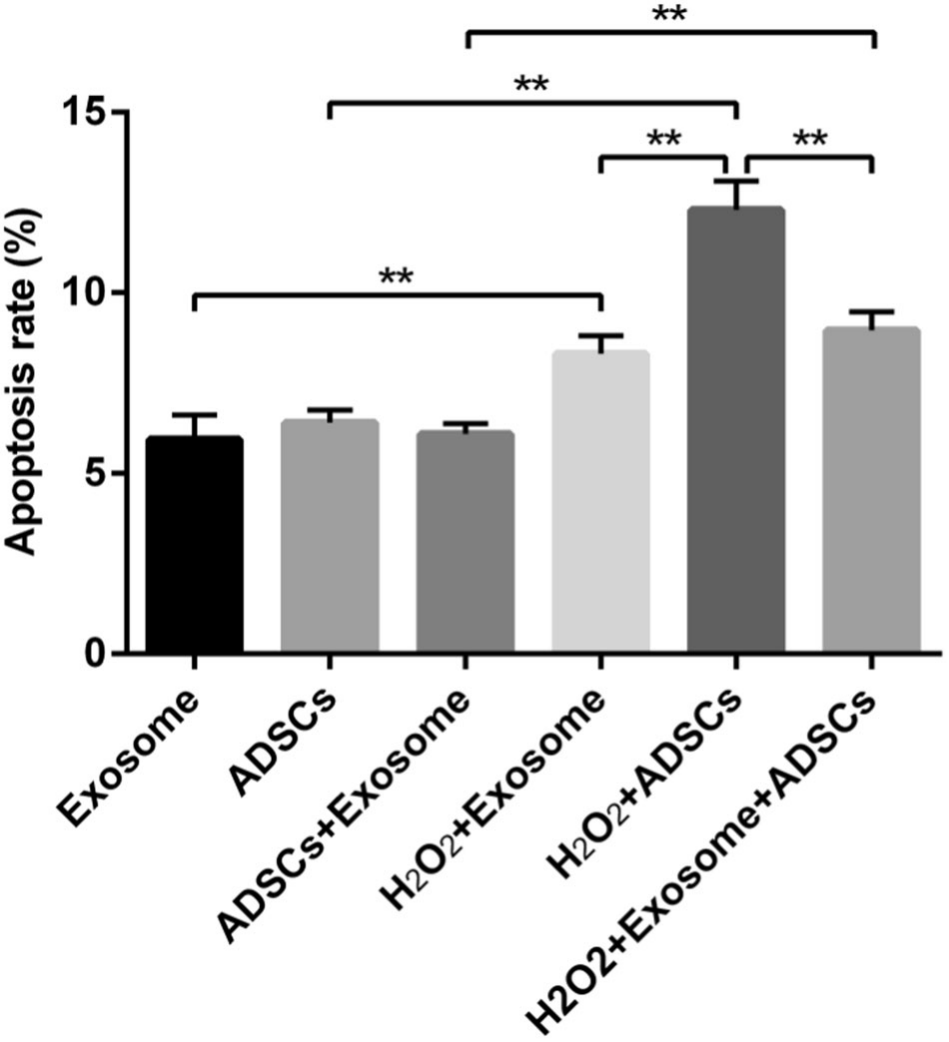
The anti-apoptotic effects of ADSC-exosomes on MCM cells after treatment with H_2_O_2_

In order to determine the effects of exosomes on oxidative stress-induced apoptosis in cardiomyocytes, MCM cells were co-cultured with ADSCs, and/or ADSC-exosomes for 24 h. Co-cultures were then treated with 0 or 200 μM H_2_O_2_ for 4 h. We measured apoptosis rates in six groups of MCM cells, as shown in Fig. 4. The apoptosis rates were similar among the exosomes, ADSCs, and all co-cultures indicating that exosomes and ADSCs did not alter apoptosis under normal conditions (Fig. 4). The number of apoptotic cells was significantly increased in H_2_O_2_-treated MCM cells. Co-culture with ADSC-exosomes significantly suppressed H_2_O_2_-induced apoptosis (*P* < 0.01). These results indicate that ADSC-exosomes protect MCM from oxidative stress.

## Discussion

Previous evidence suggests that exosomes from various stem cells can stimulate regeneration and enhance myocardial function. The present study demonstrates that exosomes from ADSCs decrease oxidative stress-induced apoptosis of myocardial cells. Exosome treatment may restore bioenergetics by complementing key enzymes in the oxidative phosphorylation pathway that were lost during myocardial ischemic reperfusion injury. Exosomes can increase levels of adenosine triphosphate (ATP), reduced nicotinamide adenine dinucleotide (NADH), phosphorylated-Akt, and phosphorylated-GSK-3β, and reduce phosphorylated-c-JNK in hearts subjected to ischemic/reperfusion^12^.

Transfer of RNA and DNA through exosomes has emerged as a crucial mechanism by which exosomes can elicit cardioprotection. Genetic exchange can induce gene changes in nearby cells^27,28^. For example, miRNAs can directly regulate the stability of other RNAs and participate in transcriptional regulation of many genes. MiRNAs control important pathological outcomes in myocardial infarction, including the inhibition of myocardial cell apoptosis. MiR-22, which is enriched in MSCs following ischemic preconditioning, has an antiapoptotic effect mediated by direct targeting of methyl CpG binding protein 2^29^. MSC overexpression of miR-221 significantly enhances cardioprotection by reducing the expression of p53-upregulated modulator of apoptosis (PUMA)^30^. After AMI, CD34^+^ cells release exosomes enriched with miRNA-126, which promotes angiogenesis^31,32^.

Protective functions of exosomes have also been attributed to heat shock proteins (HSPs). HSPs can repair ion channels, restore redox balance, interact with nitric oxide-induced protection, inhibit proinflammatory cytokines, and prevent apoptosis pathway activation^33^. HSPs are intracellular chaperones important for correct protein homeostasis. Specifically Hsp70 and Hsp27 are capable of protection against irreversible injuries associated with ischemia reperfusion^34–36^. Hsp70 is secreted from cells by exosomes, and protects against oxidative stress and apoptosis; Hsp70 also maintains sarcomeric structure^35,37,38^. Vicencio showed that exosomal Hsp70 activates the downstream pathway of toll-like receptor (TLR) 4, leading to phosphorylation of Hsp27^9^. Elevated levels of Hsp27 participate in cardioprotection by maintaining the integrity of microtubules and actin cytoskeleton, and protecting the endothelium from ischemia^39,40^. Hsp27 can also act as an endogenous cytoprotective stress response protein, eliciting cardioprotection after ischemic injury via its role as a molecular chaperone^41^. Hsp27 plays a role as a downstream effector of p38 MAPK during ischemic or β-adrenergic preconditioning protocols^42^. The Hsp70–TLR4–Hsp27 axis is one of the critical components in exosome-mediated cardioprotection.

Exosomes from ADSCs promote neovascularization and alleviate inflammation and apoptosis after ischemia reperfusion^43^. Treatment of macrophages with ADSC-exosomes induces the anti-inflammatory M2 phenotype through transactivation of arginase-1 by exosome-carried active signal transducer and activator of transcription^44^. Luo demonstrated that exosomes from miR-126-overexpressing ADSCs decreased H9c2 myocardial cell injury by reducing inflammation factor expression during hypoxia induction^45^.

Exosomes may also have a therapeutic effect by reprogramming the microenvironment. Exosome injections significantly reduced apoptosis, in rats that underwent ischemia reperfusion, by enhancing autophagy via the AMPK/mTOR and Akt/mTOR pathways^22^. IL-6, secreted from ADSC-exosomes, stimulated angiogenesis and enhanced recovery after ischemia reperfusion injury by the classic signaling pathway^46^.

In summary, a variety of stem cell exosomes can protect against ischemia injury in the myocardium. This study confirms that exosomes originating from ADSCs can protect cardiomyocytes from oxidative stress.

### Study limitations

It is only an experiment in vitro. Future experiments will be in vivo.
